# Artificial intelligence in healthcare: combining deep learning and Bayesian optimization to forecast COVID-19 confirmed cases

**DOI:** 10.3389/frai.2023.1327355

**Published:** 2024-01-11

**Authors:** Areej Alhhazmi, Ahmad Alferidi, Yahya A. Almutawif, Hatim Makhdoom, Hibah M. Albasri, Ben Slama Sami

**Affiliations:** ^1^Medical Laboratories Technology Department, College of Applied Medical Sciences, Taibah University, Al-Madinah Al-Munawarah, Saudi Arabia; ^2^Department of Electrical Engineering, College of Engineering, Taibah University, Al-Madinah Al-Munawarah, Saudi Arabia; ^3^Department of Biology, College of Science, Taibah University, Al-Madinah Al-Munawarah, Saudi Arabia; ^4^Computer Sciences Department, The Applied College, King Abdulaziz, Saudi Arabia University, Jeddah, Saudi Arabia

**Keywords:** artificial intelligence, algorithm, Bayesian optimization, COVID-19, deep reinforcement learning, decision-making, healthcare, prediction

## Abstract

Healthcare is a topic of significant concern within the academic and business sectors. The COVID-19 pandemic has had a considerable effect on the health of people worldwide. The rapid increase in cases adversely affects a nation's economy, public health, and residents' social and personal well-being. Improving the precision of COVID-19 infection forecasts can aid in making informed decisions regarding interventions, given the pandemic's harmful impact on numerous aspects of human life, such as health and the economy. This study aims to predict the number of confirmed COVID-19 cases in Saudi Arabia using Bayesian optimization (BOA) and deep learning (DL) methods. Two methods were assessed for their efficacy in predicting the occurrence of positive cases of COVID-19. The research employed data from confirmed COVID-19 cases in Saudi Arabia (SA), the United Kingdom (UK), and Tunisia (TU) from 2020 to 2021. The findings from the BOA model indicate that accurately predicting the number of COVID-19 positive cases is difficult due to the BOA projections needing to align with the assumptions. Thus, a DL approach was utilized to enhance the precision of COVID-19 positive case prediction in South Africa. The DQN model performed better than the BOA model when assessing RMSE and MAPE values. The model operates on a local server infrastructure, where the trained policy is transmitted solely to DQN. DQN formulated a reward function to amplify the efficiency of the DQN algorithm. By examining the rate of change and duration of sleep in the test data, this function can enhance the DQN model's training. Based on simulation findings, it can decrease the DQN work cycle by roughly 28% and diminish data overhead by more than 50% on average.

## 1 Introduction

### 1.1 Overview and research context

COVID-19 has impacted people globally since its discovery (Yoshikura, 2022). It has strained healthcare systems and economies, leading multiple countries to implement extensive measures to mitigate the pandemic's effects (Reshi, 2020). While these interventions vary by country, typical strategies involve social distancing, border closures, school closures, and prohibiting public gatherings (Khetarpaul, 2021). The techniques utilized by 11 European nations have effectively decreased the dissemination of COVID-19 (Clay-Wililams et al., 2020). With the pandemic's wide-ranging impact and no existing cure, it is crucial to evaluate the number of probable cases (McFee, 2020).

Several methods, including recurrent neural networks (RNNs), gated recurrent units (GRUs), long short-term memory (LSTM) networks, graph neural networks (GNNs), and others, have been utilized to forecast infectious diseases (Li and Sun, 2022). Therefore, computational and computational intelligence models could be employed to predict COVID-19 situations. Prior studies have established that prediction models based on neural networks (NNs) can provide accurate forecasts (Yu et al., 2021). Therefore, neural networks possess considerable potential for investigating the epidemiology of viruses. Neural networks (NNs) have been utilized to forecast essential factors such as the prevalence of cases, mortality rates, immunization rates, extreme poverty levels, accessibility of hand-washing facilities, weekly hospitalizations, weekly hospitalizations per million people, ICU patients per million people, hospitalized patients per million people, and the concentration of patients in ICUs. The research by Haque et al. (2022) provide a comprehensive review into the prediction models that have been introduced. It investigates how well neural networks (NNs), long short-term memory (LSTM) models, and fully connected transformer models can predict the spread of COVID-19. The forecast models also incorporated the latest data on vaccination rates per million, total vaccination figures per hundred, and the most recent vaccination data (Shrestha et al., 2021). Various factors can influence the manifestation and transmission of diseases. For example, disease symptoms can exhibit differences based on geographic region or country. Furthermore, a high urban population density and effective transportation networks can hasten the disease's spread (Imdad et al., 2021).

The accessibility of publicly available medical imaging datasets has resulted into a significant rise in the employment of artificial intelligence (AI) models for medical diagnosis, specifically in radiology (Nair, 2021). The ability to independently classify and generalize incoming data is a crucial determinant of such AI models' practical applicability and value. The successful deployment of trained AI models in various contexts fundamentally hinges on their generalizability. Ensuring their use remains pertinent within the intended context is crucial (Eche et al., 2021). When AI models are trained on patient groups and disease traits that are very similar to those in the training dataset, they are better at making diagnoses.

### 1.2 Literature review and contributions

Several forecasting methods have been evaluated. Taimoor et al. (2022) employed an adaptive neurological fuzzy inference system (ANFIS) to accurately forecast the number of confirmed COVID-19 cases in China. Li et al. (2021) proposed a mathematical and numerical logistic model to estimate the number of COVID-19 cases in Mexico. Furthermore, fuzzy and fractal fuzzy logic usage was applied to gauge the frequency of COVID-19 cases in 10 countries, as detailed in Castillo et al. (2022). Another study recommended using artificial neural networks and fuzzy clustering to create an innovative prediction method (Azadeh et al., 2011). Employing fuzzy aggregation boosted the precision of predicting COVID-19 transmission pathways in 12 Mexican states. While long short-term memory (LSTM) has shown effectiveness in forecasting COVID-19 infections, additional deep-learning techniques that address sequential processing difficulties remain unexplored (Borges and Nascimento, 2022).

Valente and Laurini (2021) used LSTM-based models, including ConvLSTM, BiLSTM, and M-LSTM, to examine COVID-19 transmission. Garg et al. (2022) trained Recurrent Neural Networks (RNNs), Long Short-Term Memory (LSTM) models, Bidirectional LSTMs (Bi-LSTMs), and Gated Recurrent Units (GRUs) using daily COVID-19 data. These models provided accurate new-situation forecasts. The RNN, Stacking, Bi-LSTM, and Gyrus LSTM models offered accurate estimates for the quantity of COVID-19 incidences and fatalities anticipated in the subsequent month. The investigation by Md Saleh et al. (2022) employed the LSTM model to scrutinize regularly publicized cases at national and regional levels in Spain. Dairi et al. (2021) presented the integration of Bayesian optimization, long short-term memory (LSTM), and convolutional neural network (CNN) that demonstrated commendable efficiency in predicting epidemics. A study employing LSTM-based methodology has demonstrated the potential to anticipate occurrences of COVID-19 cases and fatalities by utilizing statistical models that incorporate autoregressive integrated moving average (ARIMA), support vector regression (SVR), and deep learning algorithms, including LSTM and Bi-LSTM (Quilodrán-Casas et al., 2022).

To guarantee that all predictive models are fit for research comparisons, it is essential to ensure prediction accuracy, as MAE, RMSE values, and predicted errors depend on the study's size (Saha et al., 2022). The Bi-LSTM algorithm exhibited exceptional performance concerning mean absolute error (MAE) and root mean squared error (RMSE), obtaining values of 0.0070 and 0.0077, respectively, the lowest among all algorithms. Zarzycki and Ławryńczuk (2022) explain that the architecture relies entirely on the network to use an LSTM layer. Jiang et al. (2022) proposed the incorporation of hourly weather measurements as data references in the LSTM architecture. In their 2022 study, Kistenev et al. used unbalanced microcomputed tomography (Micro-CT) imaging, supervised deep learning, and adaptive self-degradation. The ARIMA method has been utilized in recent research (Chyon et al., 2022; Garg et al., 2022; Zarzycki and Ławryńczuk, 2022). The implementation of artificial neural networks (ANN) and long short-term memory (LSTM) techniques has improved the precision of time-series prediction studies for COVID-19 (Box, 2018). Implementing approaches based on fuzzy logic has also received support (Li et al., 2021). Aslan et al. (2022), Chyon et al. (2022), and Garg et al. (2022) serve as examples of how the use of advanced deep learning algorithms has improved COVID-19 prediction. Due to their sequential processing requirements, numerous deep learning models can predict COVID-19 time series data. Machine learning and deep learning are particularly well-suited for time series prediction since they can expose nonlinear patterns. Despite the potential demonstrated by attention mechanisms and convolutional neural networks, they have not been employed in the resources used for forecasting COVID-19. Using large databases, Sulthana et al. (2021) and Ma et al. (2022) developed deep-learning algorithms to predict COVID-19 cases.

Anticipating COVID-19 infections is crucial to comprehending disease transmission and making informed decisions about preventive measures (Greco et al., 2021). Technical terms are explained when first used to ensure clear communication and better understanding, and objective language is employed throughout the paper. The study compared the prediction capabilities of regression models and statistical machine-learning models. The dataset used in the research encompassed the total number of COVID-19 cases in Saudi Arabia. The study offered predictions for 1, 3, and 6 days ahead. The efficacy of BOA and DL predictions was evaluated utilizing COVID-19 case datasets from Al-Madinah Al-Munawarah, Riyadh, and Jeddah, localities with elevated COVID-19 incidence.

The study employed a stacked learning model and Gaussian processes (GP) for ensemble learning and the meta-learning model (a resilient model). We used the percentage improvement index, mean absolute errors (MAEs), and symmetric MAEs to determine how accurate each model's predictions were for missing data from the original sample. Our research analyzed various forecasting techniques for the overall number of verified COVID-19 cases in Saudi Arabia. We utilized cutting-edge machine learning models and BOA to offer crucial insights for informed decision-making regarding epidemiological management and healthcare system policies. The study looked at multi-day forecasting models that made predictions for 1, 3, and 6 days. This allowed the prediction models to be tested in various situations, which improved COVID-19 response plans. The improved strategies proposed for tackling COVID-19 have been demonstrated to be more systematically efficient than conventional and mathematical methods (as shown in Table 1).

**Table 1:** Factors addressed in various COVID-19 methods.

**Types/methods**	**Conventional COVID method**	**Advance COVID method**	**Efficiency**	**References**
ANFIS	✓	✓	°	Yoshikura, 2022
	✓	✓	°	Taimoor et al., 2022
	°	✓	°	Sulthana et al., 2021
	°	✓	✓	Greco et al., 2021
	✓	°	°	Ma et al., 2022
	°	✓	✓	Kistenev et al., 2022
	°	°	°	Dairi et al., 2021
Deep-learning	✓	°	°	Yoshikura, 2022
	°	°	°	Valente and Laurini, 2021
	°	°	✓	Quilodrán-Casas et al., 2022
	✓	✓	✓	Zarzycki and Ławryńczuk, 2022
	✓	✓	°	Li et al., 2021
Mathematics	✓	°	°	Yoshikura, 2022
	°	✓	°	Borges and Nascimento, 2022
	✓	✓	✓	Castillo et al., 2022
	°	✓		Chyon et al., 2022
Machine learning	✓	°	°	Yoshikura, 2022
	°	✓		Taimoor et al., 2022
	°	✓	✓	Sulthana et al., 2021
	°	✓		Greco et al., 2021
	°	✓	°	Quilodrán-Casas et al., 2022
	°	✓	✓	Zarzycki and Ławryńczuk, 2022
	°	✓	°	Li et al., 2021
This study	✓	✓	✓	

### 1.3 Paper organization

The paper is structured as follows: The model formulation and methodology in Section 2. The methodology presents the fundamental concept of the suggested technique. The prediction method steps are outlined in Section 3, while the achieved findings are given and reviewed in Section 4. Section 5 provides the conclusion.

## 2 Methodology

### 2.1 Methods and models

Deep learning can effectively process complex and diverse input and output changes. Deep learning can effectively process complex and various input and output changes. It enables the prediction of intricate, interdependent, noisy, and multi-stage results.

**Figure 1:**
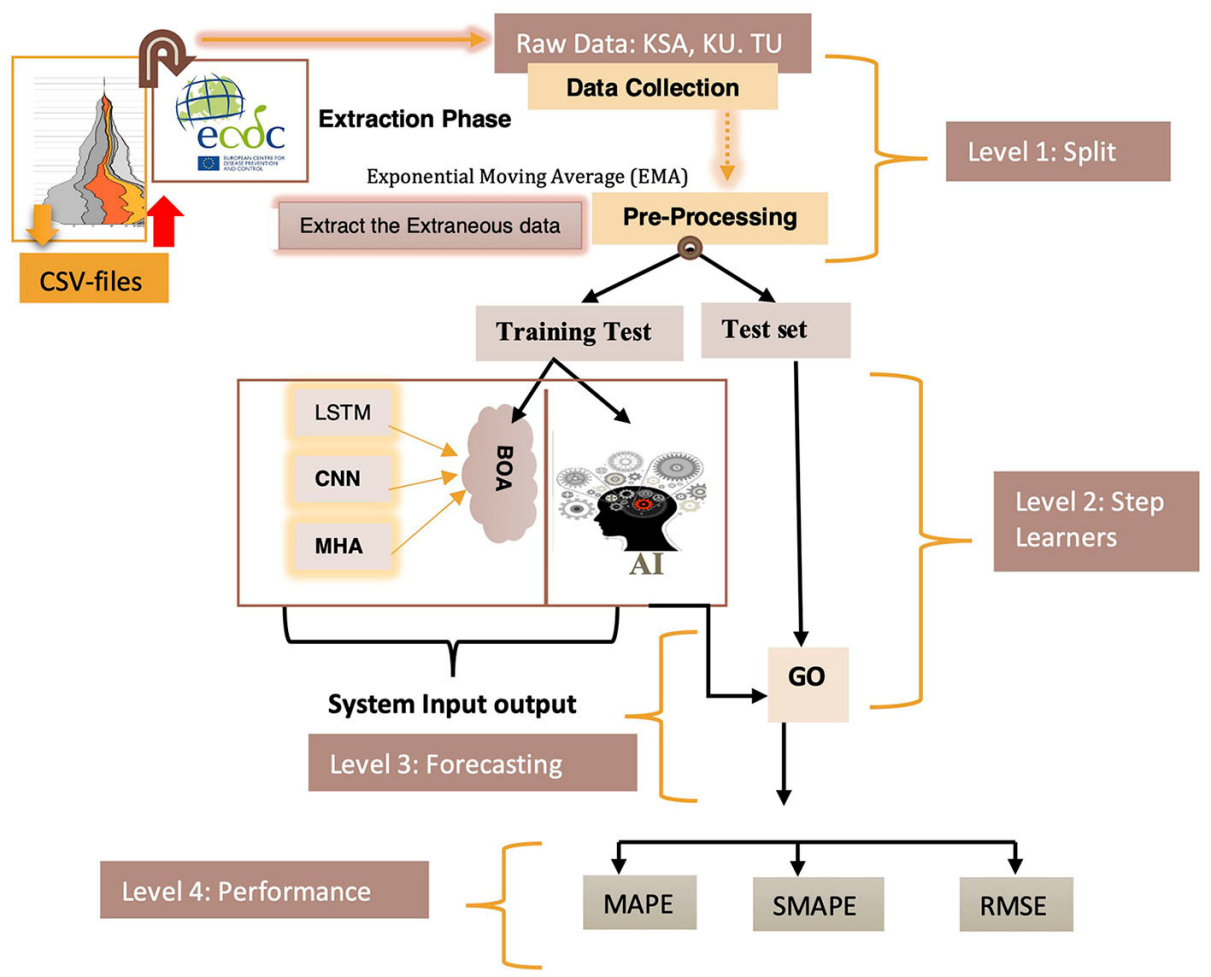
Predicted COVID-19 forecasting model using DQN approach.

The AI-driven COVID-19 prediction model displayed in Figure 1 was utilized in this study. This model comprises six distinct layers, each with a specific function and progressing through phases as outlined in Table 2. COVID-19 datasets were employed in predictive research, covering training and testing phases. The CNNs and Multi-Head Attention-LSTMs (MHA-LSTMs) were used to anticipate the total number of COVID-19 cases and deaths. Furthermore, Recurrent Neural Networks (RNNs) and Long Short-Term Memory (LSTM) models were examined, utilizing three unique activation functions.

**Table 2:** Model layer descriptions.

**Layers (Li)/phases (Pi)**	**L_1_**	**L_2_**	**L_3_**	**L_4_**	**L_5_**	**L_6_**
Split	*Extraction*	*Extraneous data*	^*^	^*^	^*^	^*^
Test	^*^	^*^	*Training test*	^*^	^*^	^*^
Step learners	^*^	^*^	^*^	*Hybrid model*	^*^	^*^
Forecasting					*Forecasting*	^*^
Performance	^*^	^*^	^*^	^*^	^*^	*Performance*

The symbol ^*^ means not cited.

The proposed prediction system is organized into four tiers, each carrying out specific tasks based on predetermined standards (Figure 1). Data collection occurred during level 1, including a training test and a test set. Step 2 involved acquiring knowledge, while levels 3 and 4 predicted and evaluated the system's effectiveness.

The COVID-19 data was utilized in predicting time series (TS) as it includes daily new case statistics. Table 3 features the TS analysis of COVID-19 assumptions and the modeling techniques employed across countries, studies, and data periods. The development of TS forecasts for COVID-19 involved different methods like statistical analysis, machine learning, deep learning, and fuzzy logic.

**Table 3:** Sleep time of training test (TT).

**Fixed action (A^e^)**	**Sleep time—TT**
0	850 s
1	410 s
2	230 s
3	60 s

Initially, the raw data was separated into training and testing data sets. The training dataset contained the most samples, while the testing dataset consisted of the six most recent observations. Following this, calculations were performed on the training data to determine the mean and standard deviation.

A recursive approach was used to forecast COVID-19 cases with a lead time of several days. The model initially predicted the number of cases for the following day. The forecast for the next day was determined through an iterative process in which the expected value obtained from the model was reinserted into the same model. This methodology was repeated until the intended outcome was achieved. The training approach utilized in this research can be outlined as follows (see Equation 1) (Su et al., 2022):


(1) $$\{\begin{array}{l} x(t+1)=g\left\{x_{t},\ldots ..x_{t+1-\sigma }\right\}+\xi \\ \xi \in M(D=0,\delta^{2}) \end{array}$$

In this equation, ***g*** represents the function derived from the model used during the learning phase, ***x*****_t+1_** signifies the COVID-19 cases forecasted 1 day, **δ** is the number of confirmed cases from the past (δ = 5), and **ζ** denotes a random error conforming to a normal distribution with a mean (***D*** **=** **0**) and variance (**σ^2^**).

The study explored instances looking ahead up to ***M*** days, specifically up to 1, 2, and 6 days. These selected time frames are detailed as follows:

a. Day One Test (**DOT**).
b. Day Three Test (**DTT**).
c. Sixth day -Test (**DST**).

The following *x*_t+1_ structures were evaluated as follows (see Equation 2) (Yu et al., 2021):


(2) $${\bar{x}}_{t+k}=\{\begin{array}{l} \bar{g}\left\{x_{t},x_{t-1}\ldots ..x_{t-\sigma +1}\right\}-->k=1\\ \bar{g}\left\{{\bar{x}}_{t+k-1},x_{t+1},x_{t},\ldots ..x_{t+k-\sigma }\right\}-->k\in \left[2,\sigma \right]\\ \bar{g}\left\{{\bar{x}}_{t+k-1},\ldots .x_{t+k-\sigma }\right\}-->k\in \left[\sigma +1,M\right] \end{array}$$

## 3 Prediction method steps

### 3.1 Bayesian optimization

The above-mentioned equation utilized BOA (Bayesian definition in Supplementary Appendix 1), which employed the pre-distribution function g(w) and sample information to calculate the suffix. The resulting suffix then determines the optimal parameter value for the task (Aslan et al., 2022). The acquisition function is a mathematical expression that represents the criteria for BOA (*z*). The value function (*z*), also referred to as an acquisition function, acts as the deciding factor for choosing the subsequent sample point that maximizes the utility function (see Equation 3) (Fanelli et al., 2021):


(3) $$\begin{array}{l} \begin{array}{c} {w_{t}}^{+}=\arg\underset{w\in S}{\max}z\left(w|{J\left(t\right)}_{1:t-1}\right)\\ {J\left(t\right)}_{1:t-1}={\left\{x^{n},y^{n}\right\}}_{n=1}^{t=1} \end{array} \end{array}$$

Algorithm 1 represents BOA, indicating the training dataset of the ***t***–**1** factor for function g. The algorithm can be divided into two parts: the post-distribution update (**steps 4 and 5**) and the maximization of the Acquisition Function (**Step 3**) (Garg et al., 2022).

**Algorithm 1:**
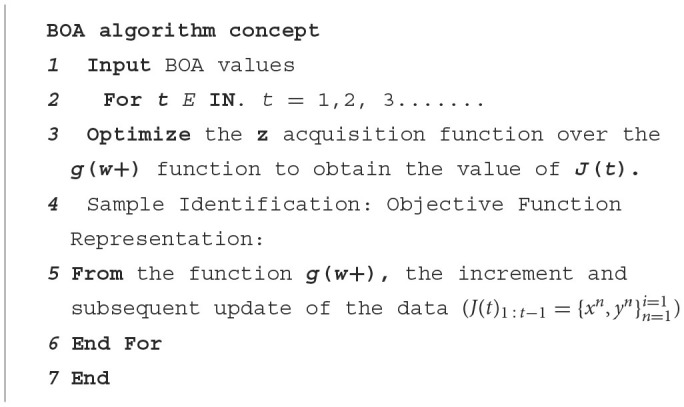
BOA algorithm.

#### 3.1.1 Gaussian process

A Gaussian process (GP) was created using a stochastic GP and Bayesian learning theory, expanding our understanding of the concept of a Gaussian probability distribution. Unlike the probability distribution, the random process establishes the properties of the functions (Aslan et al., 2022). The GP consisted of random variables that adhered to a Gaussian distribution. Only the average and covariance functions of these variables could determine their characteristics. A Gaussian process with a linear kernel was employed in this study and named accordingly (Taimoor et al., 2022). When a Gaussian stochastic method comprises more than two variables, any finite subset of random variables conforms to a Gaussian distribution (see Equation 4) (Taimoor et al., 2022):


(4) $$|\begin{array}{l} g(w^{+})\approx E(h(w^{+}),I(w^{+},w^{t}))\\ I(w+,w^{t})=Exp\left[-\frac{1}{2}{\left\|w_{i}-w_{j}\right\|}^{2}\right] \end{array}$$

Here, *w*_i_ and *w*_j_ represent the *i*th and *j*th samples, respectively. They converge to 1 when the two samples are identical, and to 0 otherwise. When the two sampling points are close, they exhibit a strong correlation and mutual influence, while they have a weaker influence when they are distant from each other. The posterior distribution of *g*(*w*^+^) is defined as follows (see Equation 5). Commencing with the training set, *z*(*w*^+^) is observed, and *g*(*w*^+^) is drawn from a multivariate normal distribution (Garg et al., 2022).


(5) $$I(w^{+},w^{t})=\left[\begin{array}{cccc} I(w_{1},w_{1}) & I(w_{1},w_{2}) & \ldots . & I(w_{1},w_{t})\\ I(w_{2},w_{1}) & I(w_{2},w_{1}) & \ldots . & I(w_{2},w_{t})\\ \ldots & \ldots & \ldots & \ldots .\\ I(w_{n},w_{1}) & I(w_{n},w_{1}) & \ldots . & I(w_{n},w_{t}) \end{array}\right]$$

The elements ***I*****(*w*^+^**, ***w*****^t^)** were computed as outlined in Equation 3. ***I*****(*w*^+^**, ***w*****^t^)** represented the sample estimate while excluding the noise, specifically the diagonal component. BOA successfully determined the optimal value with only a few samples, eliminating the need for expressing the function as required by typical optimization methods. BOA was particularly well-suited for hyperparameter adjustments. In this section, hyperparameters for the machine learning models (MHA, LSTM, and CNN: Supplementary Appendixes 2, 3) were optimized using BOA. The random forest (RF) served as a conventional method for predicting clustering performance (Supplementary Appendix 2).

### 3.2 Deep learning approach

#### 3.2.1 Problem formulation using a Markov decision process

The Markov Decision Process (MDP) outlines environments for Reinforcement *Q*-Learning (RL), dealing effectively with uncertain outcomes. The MDP for an agent in a specific domain is shown as a tuple (S^e^, A^e^, P^e^, R^e^), where S^e^ is the set of possible problem states, Ae is the set of possible actions, Pe is the chance of going from state s^a^ to state s' through an action (a, A^e^), and Re is the immediate reward that the agent gets by doing an action in state s and going from state s^a^ to state s′ (see Equation 6) (Azadeh et al., 2011).

**Agent (A**^**e**^**)**: this representation thus encompasses the agent (A^e^). The proposed approach's energy conditions and the environmental light intensity were considered input training test (TT) data by this program. The policies established by the DQN were updated, and BN sleep time was used as an example of the optimal approach.

**Environment (E**^**e**^**)**: anything unrelated to the agent, such as other approaches, events, and wireless channels, fell under the environment (E^e^).

**State (S**^**e**^**)**: the deep learning model proposed in this study adjusts its mode and activity by collecting data and utilizing or acquiring energy. This article describes the methodology for each input training test as follows:

d. Training Test (TT)
e. Day One Test (DOT)
f. Day Three Test (DTT)
g. Sixth day-Test (DST)
h. Time (*t*)
i. The Rate of Change (RoC) of the TT detected data.

(6) $$\begin{array}{l} R^{e}\left(w\right)=w*|1-w\left(\frac{A^{e}}{3}\right)|+\left(1-w\right)*\left(1-|D^{r}-\left(\frac{A^{e}}{3}\right)|\right) \end{array}$$

**Action (A**^**e**^**)**: the duration of sleep for an approach labeled “active” was determined for each state based on Equation 6, which remained constant. The work cycle depended on the recipient's sleep duration, between 1 and 900 seconds. Four suggested measures are displayed in Table 2.

**State transition [S**^**e**^ **(s**^**a**^**, s**′**)]**: was characterized by multiple stages within a 24-h episode. Transmitting a state necessitated a short time interval, resulting in elevated communication overhead and energy usage. Applying extended time intervals would erroneously alter the duration of sleep and the frequency of measurements. Studies have demonstrated that a time interval of 15 min achieved a satisfactory balance between the quality of service and the amount of communication required. Consequently, the time interval was established to be 15 min. The proposed approach entailed regular reporting of state changes to the base stations every 15 min. The agent was tasked with determining sleep duration during the training phase and transmitting a behavioral policy.

**Reward (R**^**e**^**)**: the sleep duration for the training assessment was modified under the time of the total evaluation (Re). Previous studies exclusively examined the magnitude of the assessment data component. However, if the rate of change in the training test data was low, the number of transmissions decreased, or the duration of the training test sleep period was extended, two perpendicular goals were considered: rate of change and sleep time. Equation 8 used the reward function to achieve this goal.

In this context, “*w*” denotes the standardized level of the TT quantity that ranges from 0 to 1. The action index pertains to the specific index linked with the action performed, i.e., the chosen duration of sleep, as explained in Table 3. “Dr” indicates the rate of change of the analyzed values in the preceding phase. The reward function is based on the rate of change in the data of the suggested technique, sleep duration, and TT level.

#### 3.2.2 DQN structure

The DQN architecture comprised a primary and a secondary network with corresponding functionalities. The primary network was responsible for assessing the *Q*-value of probable state actions (*Q*-value prediction) and calculating the *Q*-value of each state. Following the stabilization of the primary network, adjustments were made to the target network's features. Following the stabilization of the primary grid, the parameters of the secondary network were modified to calculate the *Q*-value for the target network—the optimal *Q*-value for the specific condition.

**Figure 2:**
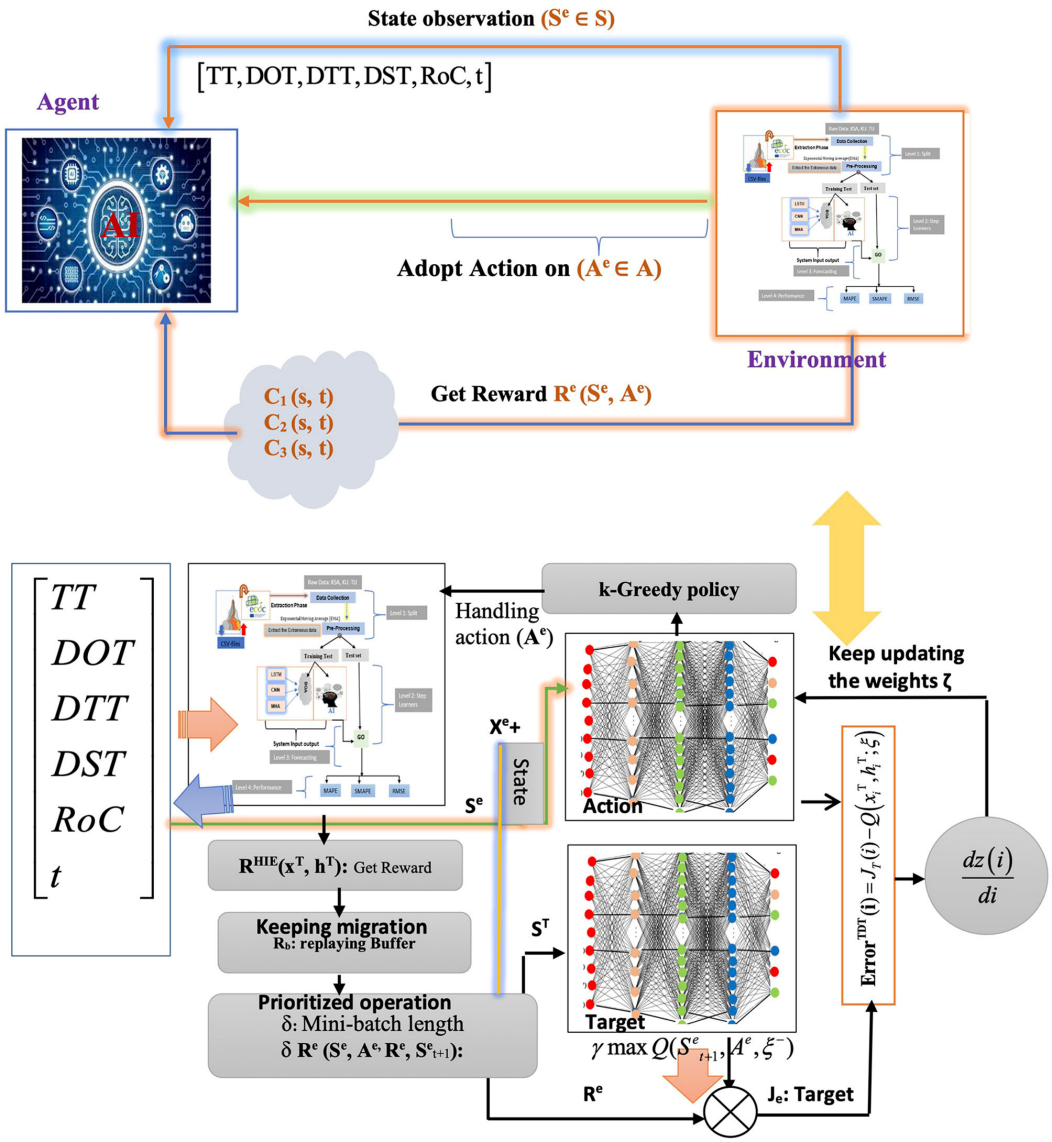
DQN-based management approach.

Figure 2 illustrates the functionality of DQN as a reinforcement learning algorithm, enabling the agent to operate as such. As a reinforcement learning algorithm, DQN allows the agent to gain knowledge by repeatedly testing different behaviors in various states and discerning the most favorable ones while interacting with the environment. Throughout each stage of the episode, the agent acted in S^e^ states **S**^**e**^ **(s**^**a**^**, s**′**)**, incorporating the continuous values of the feature vector that demonstrate the agent's state, such as the level of TT charge, the intensity of environmental light on various days of the week, and the rate at which TT values changed. The agent procured a multitude of experiences (**S**^**e**^**, A**^**e**^**, P**^**e**^**, R**^**e**^), encompassing sensory experiences (S^e^), action experiences (A^e^), perceptual experiences (PE), and reflective experiences (R^e^). The epsilon-greedy policy was employed to choose actions, similar to the *Q*-learning approach. Nevertheless, instead of using a *Q*-array, the central neural network evaluated the *Q*-values of the actions (refer to Equation 7) (Aslan et al., 2022).


(7) $$\{\begin{array}{l} L(\theta )=\frac{1}{n}\sum_{i=1}^{k}{\left(z_{i}-Q_{\theta }(S^{e},A^{e})\right)}^{2}\\ z_{i}=\{\begin{array}{l} R^{e}(i)-->step(i)+1\\ R^{e}(i)+\gamma \max\bar{Q}(S^{e}(i+1),a,\bar{\theta }) \end{array} \end{array}-->$$

Here, *k* signifies the dataset size, and ***Q*****(s**_**i**_**, a**_**i**_**)** indicates the estimated *Q*-value from the primary network for action **a**_**i**_ in state **S**^**e**^ **(s**_**i**_**)**. The *z*_i_ was determined using Equation 7 was regarded as **R**^**e**^**(i)** if the following statement was final. If **R**^**e**^**(i)** was unknown, z_i_ was calculated using the target network to estimate the Q-values of potential actions in the state Se (i + 1). $S_{\text{i+1}}^{\text{e}}$ was assessed in this state via the target network, which led to the reward Re(i) evaluation as the product of the maximum *Q* value and the discount factor. This indicates that the reward factor **R**^**e**^**(i)** results from multiplying the maximum *Q* value by the discount factor. During each time step, the primary neural network's parameters were updated using gradient descent, with target network updates occurring only at each TT time step. As a result, the primary network's parameters were replicated onto the target network, with multiple iterations completed to achieve the optimal *Q*-value.

### 3.3 The proposed approach

**Figure 3:**
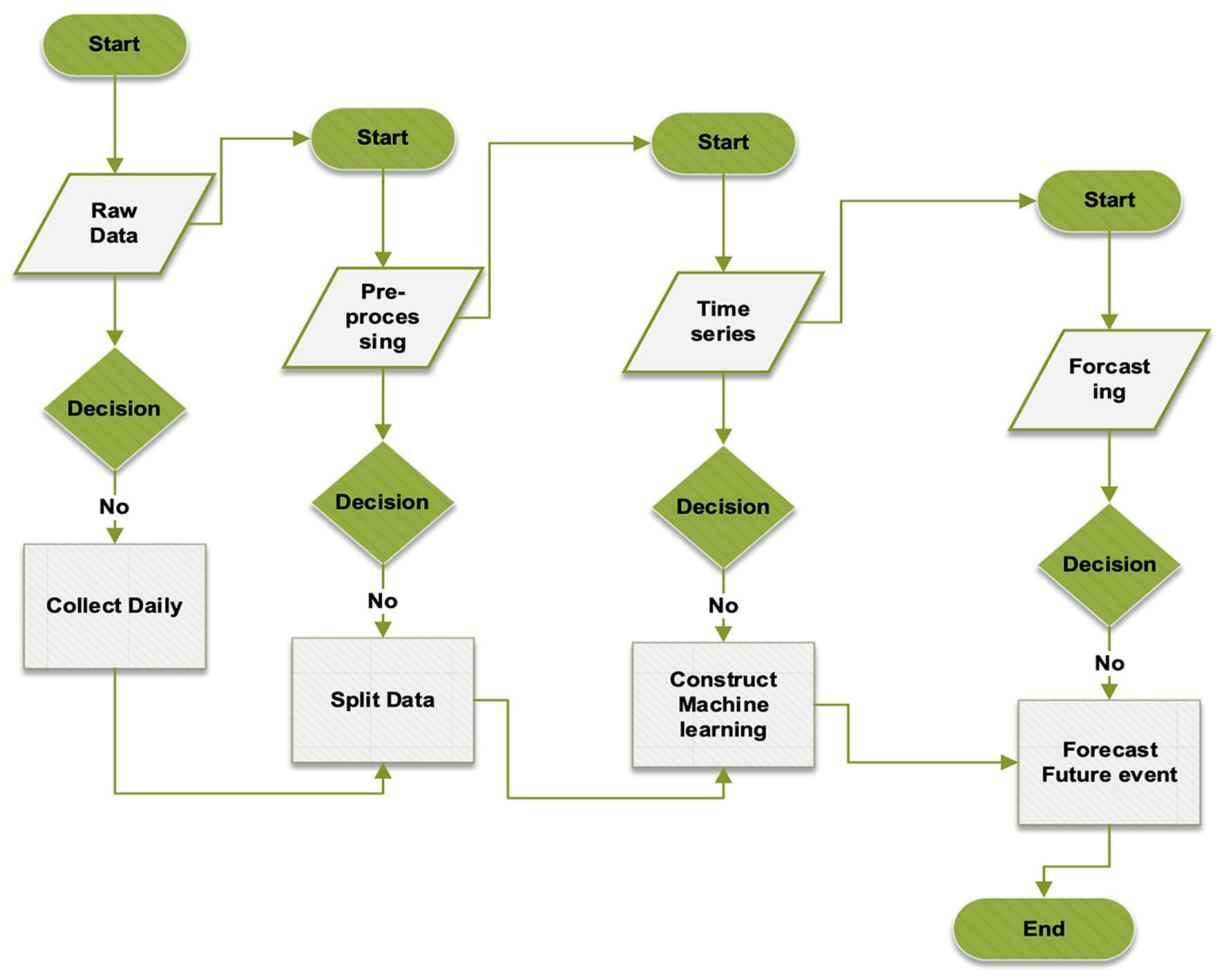
Proposed forecasting models.

This study employed three approaches, namely MHA, LSTM, and CNN, to predict the cumulative number of cases. The proposed method utilized BOA to select hyperparameter settings, as illustrated in Figure 3. Furthermore, Figure 5 depicts the precise identification of hyper-optimal parameters using the BO optimizer. BOA relied more heavily on classification methods and demonstrated superior accuracy in identifying hyper-ideal parameters than RF. BOA has significantly improved matrix analysis. The Dynamic Link Aggregation (DLA) is enabled when the test condition (*L* < δ) is met. Algorithm 2 outlines the DQN technique for adjusting the duty cycle of TT collection.

**Algorithm 2:**
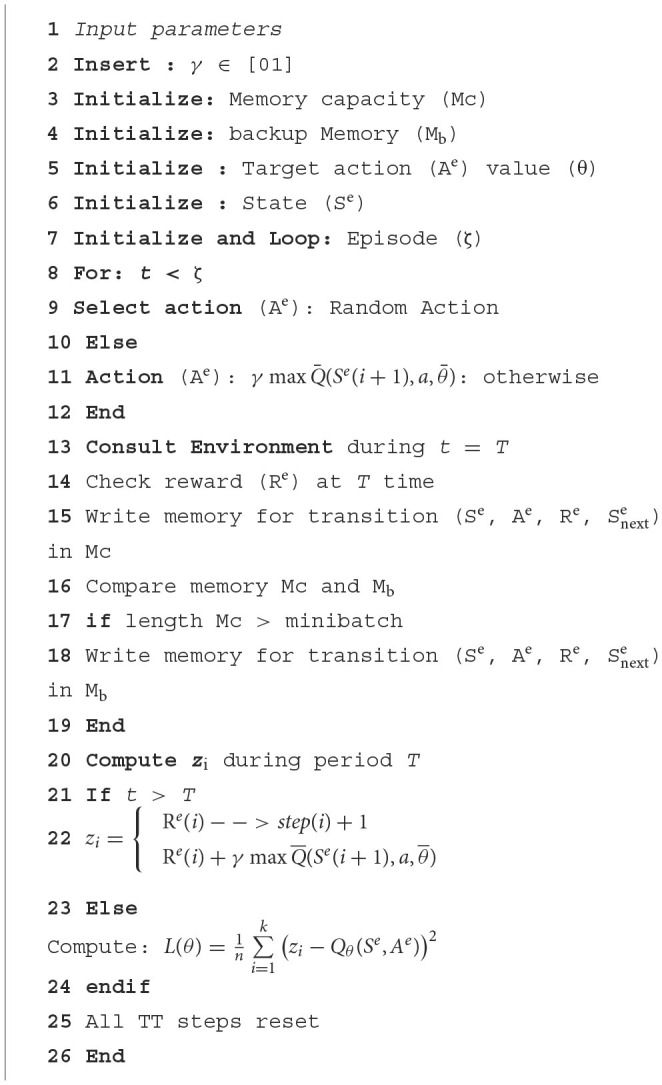
Proposed forecasting approach.

The algorithm's hyperparameters were first retrieved to create a replay memory using M bits. Subsequently, the primary network parameters were established, with identical settings replicated for the target network. The neural network underwent several Time-Triggered (TT) training rounds, with improved TT sleep time during specific episodes. The NN was trained using a dataset spanning 120 days. Afterward, the algorithm began a series of attacks, each lasting 20 h. The variable initialization is linked to the number of steps (*t*) assigned. During each episode, the agent initially selected an action based on a greedy strategy. If a randomly distributed value within the range of 0 to 1 were below a particular threshold, the agent would randomly explore its immediate vicinity. Alternatively, the primary neural network received the current state and produced the Q values for all possible actions. The one with the highest Q value was then selected. After the time interval *T*, TT reported on its current condition and slumber duration. The reward tuple (Se, Ae, Pe, Re) is stored in the replay memory based on the current state, next state, and action. The neural network learning commenced when the data length in the replay memory exceeded the minibatch size. Initially, a *k*-minibatch was randomly selected from the response memory. To calculate the cost function in Equations 8, 9, the value of the goal zi was obtained, as per Equation 9.

### 3.4 Performance analysis

To assess the efficacy of the proposed method for forecasting COVID-19 in time-series data, three crucial metrics (Root Mean Square Error, Mean Absolute Error, and Mean Absolute Percentage Error) were calculated. The equations for computing SMAPE, MAPE, and RMSE were obtained from Equations 8–10, correspondingly explained by Jiang et al. (2022) and Kistenev et al. (2022). The model's effectiveness was assessed through out-of-sample predictions from the test set (TS).


(8) $$\begin{array}{l} \text{SMAPE}=\frac{1}{\xi }\overset{\xi }{\underset{j=1}{\sum }}\left[\frac{\left|\hat{J_{t}}-J_{t}\right|}{\frac{{\left|\hat{J_{t}}-J_{t}\right|}^{2}}{2}}\right]*100 \end{array}$$

(9) $$\begin{array}{l} \text{MAPE}=\frac{1}{\xi }\overset{\xi }{\underset{j=1}{\sum }}\left[\frac{\left|\hat{J_{t}}-J_{t}\right|}{\left|J_{t}\right|}\right]*100 \end{array}$$

(10) $$\begin{array}{l} \text{RMSE}=\sqrt{\frac{1}{\xi }\overset{\xi }{\underset{j=1}{\sum }}|J_{t}-\hat{J_{t}}|*100} \end{array}$$

where $\hat{J_{t}}$ and terms of timing the estimated and measured amount at timestep ***t***.

## 4 Simulation results and analysis

Using sophisticated algorithms is crucial for improving the accuracy of COVID-19 predictions. This can mitigate the virus's impact on the public's well-being, the economy, and essential resources. This research demonstrates the process of predicting coronaviruses using the Deep Learning (DL) technique. The COVID-19 life cycle and transmission investigation in Saudi Arabia used online datasets and a DL methodology to compute the Root Mean Square Error (RMSE) for each country's training and testing data. The algorithm's efficacy was evaluated through trials conducted using Kaggle-collected data. The experiment assessed the effectiveness of three algorithms: BOA, and LSTM. The most suitable model was chosen based on the prediction error rate, quantified using the Root Mean Square Error (RMSE). The BOA model is regarded as the most effective for forecasting COVID-19 confirmed cases among the three models currently employed in Saudi Arabia. Subsequently, the LSTM model predicts the total number of reported and recovered cases worldwide.

### 4.1 Dataset description

**Figure 4:**
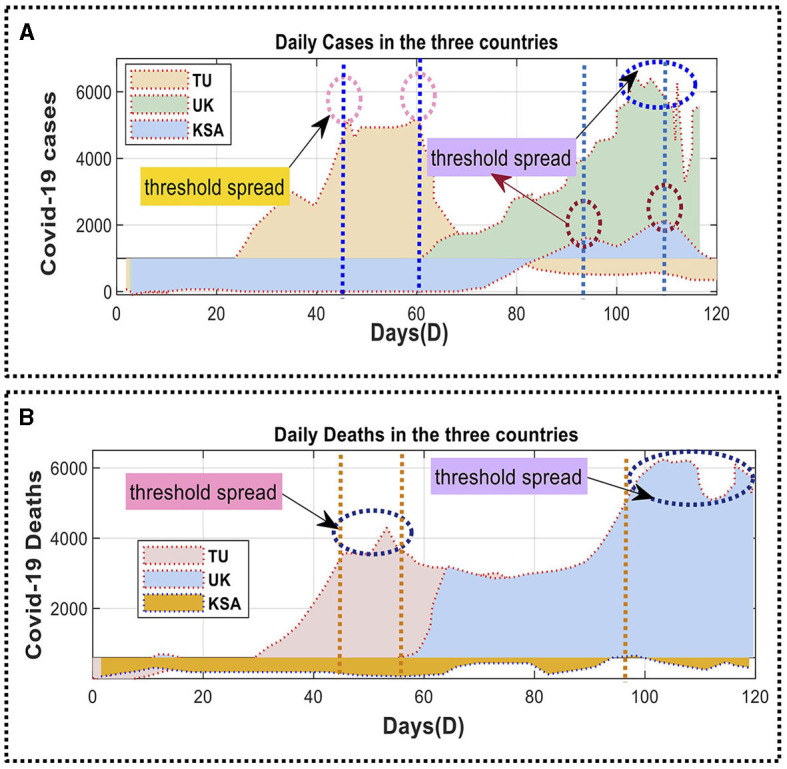
Daily confirmed deaths for COVID-19 cases in Saudi Arabia, Tunisia, and the United Kingdom: **(A)** the number of new COVID-19 cases each day; **(B)** number of new COVID-19 cases and deaths per day.

The European Center for Disease Prevention and Control (ECDC) collected and analyzed the datasets used in this study. Figure 4 illustrates the daily mortality rate and the cumulative number of confirmed infections in SA, TU, and the UK. The analysis started on April 10, 2020, and ended on June 10, 2020, over 120 days. The dataset consisted of 900 entries and had a file size of 4.8 megabytes. The dataset was split into training and evaluation sets, with around 79% allocated for training and the remaining 20% used for assessing the model parameters. This method was employed in previous studies by Box (2018), Azamifard et al. (2020), Kutlu and Camgözlü (2021), Shrestha et al. (2021), Sulthana et al. (2021), Vadyala et al. (2021), Valente and Laurini (2021), Aslan et al. (2022), Garg et al. (2022), Ghimire et al. (2022), Ma et al. (2022), and Shahidzadeh et al. (2022).

After completing the instruction phase, testing was carried out. Figure 4 illustrates the daily number of confirmed COVID-19 cases in SA, TU, and UK. Figures 4A, B provide daily mortality data for the corresponding regions. The suggested predictive model was implemented and assessed to predict the number of COVID-19 cases and fatalities in these three nations. The initial RNN model was unsatisfactory and thus replaced with an LSTM-based forecasting model. The efficacy of LSTM cells in managing information leads to improved predictions. This technique implements an LSTM model with dropout and Dense layers. The activation phase of the model utilized a corrected linear activation function and a fixed random seed, leading to enhanced accuracy of gradient points.

Table 4 concisely overviews the hyperparameters employed in the DQN and BOA models. The predictions of the DQN technique were assessed using Dataset 1, taking into account data from the preceding 120 days as a benchmark. The preliminary test findings served as the basis for developing the DQN methodology.

**Table 4:** Hyperparameters for both DQN and BOA algorithm.

**Hyper-parameter**	**Value**
**BOA model**	
CNN-kernel-size	(2,3,4,5,6) -cnn-layer 1
CNN-stride=	(1,2) -cnn-layer 1
CNN-neurons	(32,64,128,256) -cnn-layer 1
LSTM-act	fun()=(ReLU Linear Tanh)
LSTM- rate:	(0.1,0.2,0.3,0.4,0. N): Dropout–cnn-layer 1
LSTM-neurons	(32,64,128,256)
MHA-activation function	(ReLU Linear)- -cnn-layer
**DQN model**	
Q-learning rate	0.01
Initial rate	0.859
Main exploration rate	0.019
Total hidden layers	3
Neurons in layer	9
Training stage (TT)	120 days
Previous time (tp)	10 min
Episode periods	20 h
Memory type Mc/Mb	500/250
Minibatch	30
Target	280

**Figure 5:**
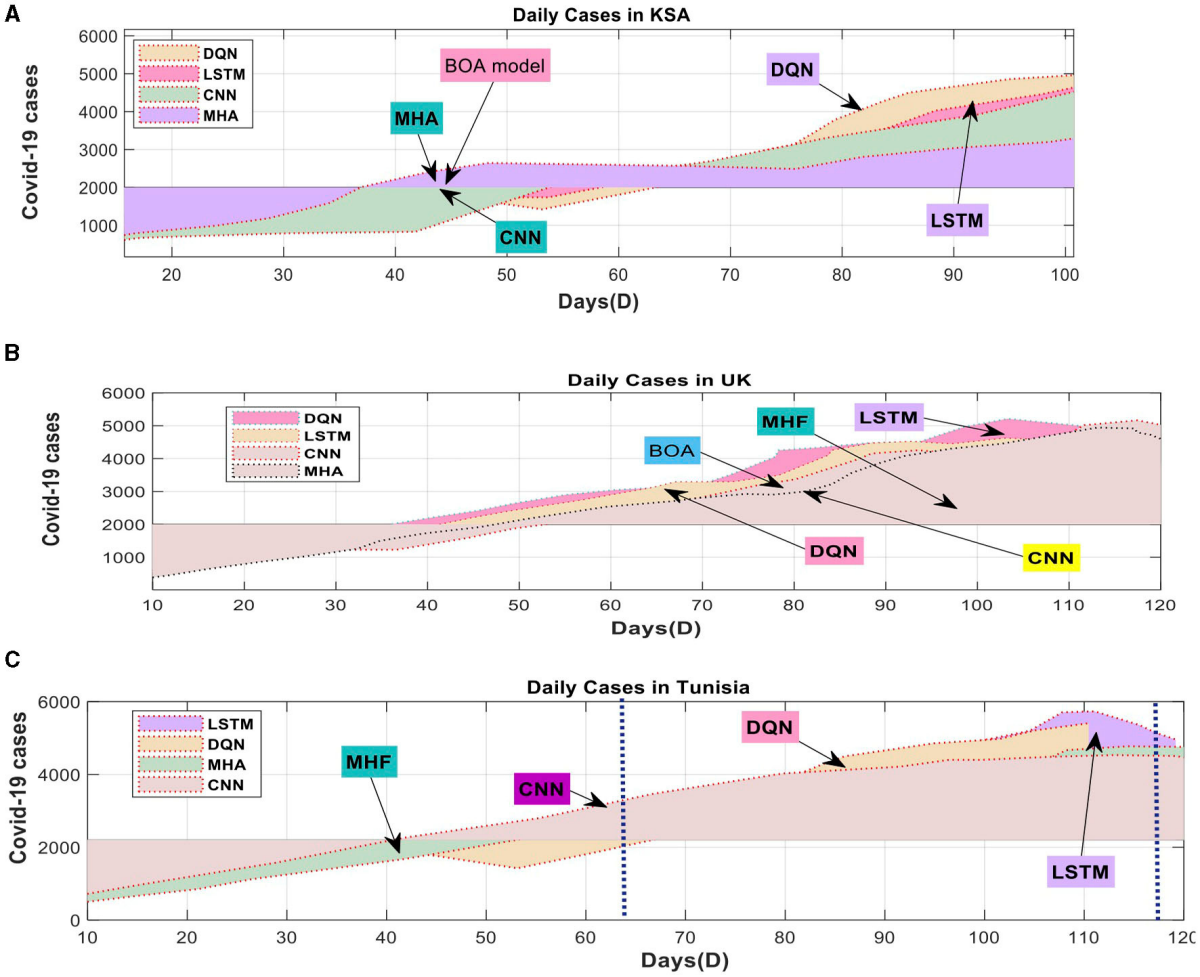
Actual (ACT) and Predicted (Pred) COVID-19 cases: **(A)** Tunisia cases, **(B)** UK cases, **(C)** KSA cases using Dataset-1.

**Figure 6:**
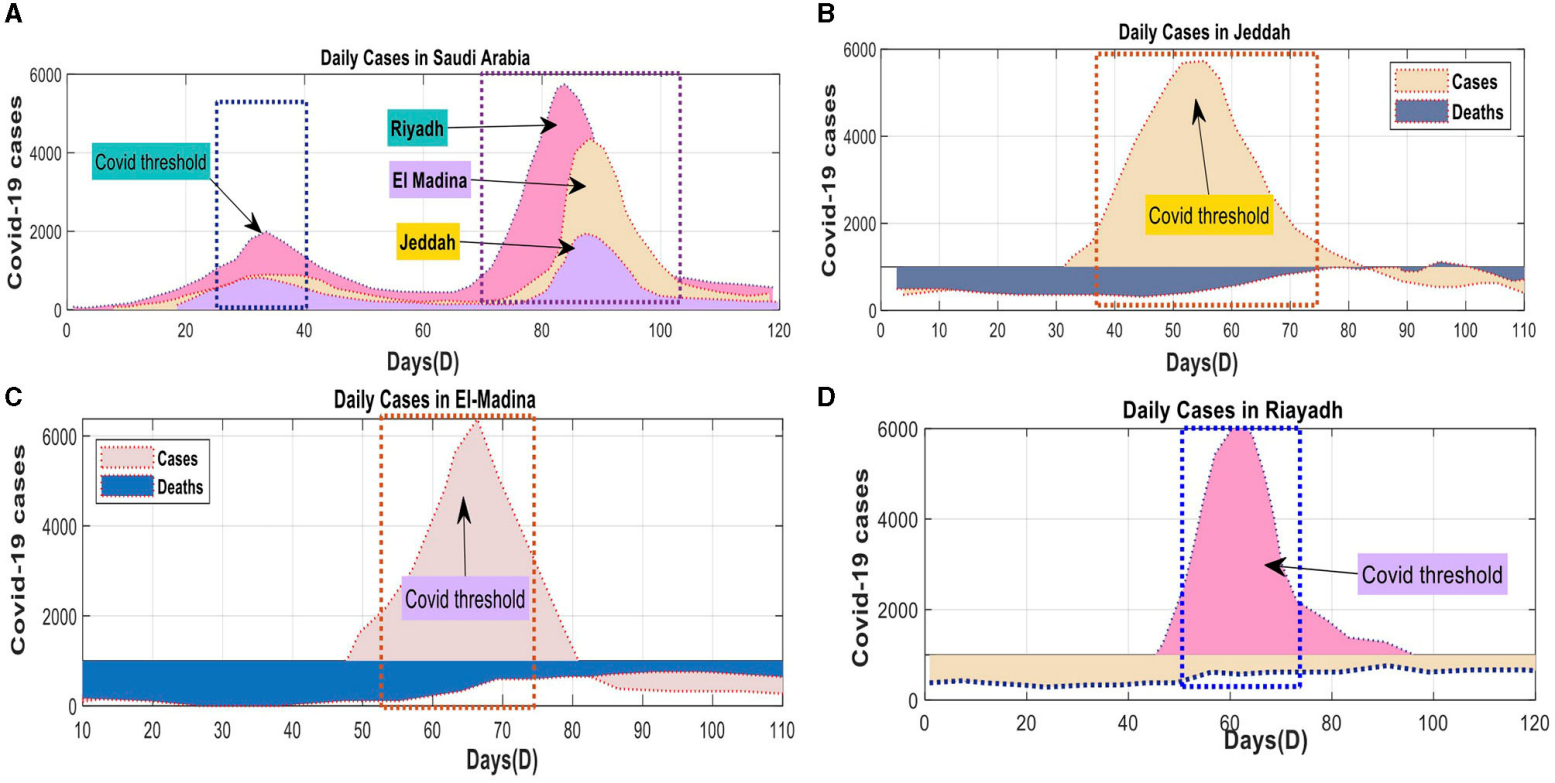
Daily confirmed COVID-19 cases and associated deaths for both KSA: **(A)** KSA Total cases, **(B)** Jeddah Province cases, **(C)** El-Madina Province cases, **(D)** Riyadh Province cases using Dataset-2.

Table 5 provides a thorough statistical summary in addition to the introduction of the suggested models for Datasets 1 and 2. Figure 5 demonstrates the strategy's efficacy by juxtaposing each nation's projected and actual instances. Figure 6A indicates that the deep learning and standard models produce disparate predictions for confirmed instances in TU. Nevertheless, the model's predictions are consistent with the actual data. Figure 5C exhibits the average number of cases in UK.

**Table 5:** Model performance based on different datasets.

**States**	**MHA**	**LSTM**	**CNN**	**DQN approach**
**Models performance relative to SMAPE**				
TU	0.054	0.061	0.062	1.0901
KSA	0.0539	0.0582	0.0993	0.0092
UK	0.354	0.530	0.358	0.5976
**Models performance relative to MAPE**				
TU	0.3687	0.4936	0.35431	0.5268
KSA	0.3985	0.568	0.4679	0.5677
UK	0.0582	0.05768	0.0524	1.1689
**Models performance relative to RMSE**				
TU	240.91	370.465	211.2 87	314.715
KSA	245.791	391.4357	213.715	314.576
UK	153.9443	198.238	119.917	199.285

In contrast, Figure 5B illustrates the anticipated values for SA, where both the deep learning and measurement models yield coherent outcomes. Within the context of TU, the conventional model exhibits slightly better performance than the deep learning model. However, on the whole, the deep learning model accurately predicts the values for the three scenarios, showing a solid agreement with the actual values. The DQN technique shows a slight superiority over the deep learning model in accurately predicting TU and SA. Figure 5A displays the anticipated values for TU. After scrutinizing the data, it became evident that the best deep learning model outperformed the DQN technique for most countries. The DQN technique regularly makes accurate linear predictions of verified cases for all regions. The model's improved predictive performance can be attributed to the deep learning system's capacity to discern linear and nonlinear data patterns. The suggested model exhibited its dependability in forecasting COVID-19 time series data.

The univariate and multivariate time series prediction models were trained using data obtained from the official website of the SA government. The Ministry of Health (MOH) provides this website as an online platform for COVID-19 statistics. The number of new cases was normalized using the highest recorded daily count of new patients. The data spanned from April 10, 2020, to June 10, 2020, and included four separate projection timeframes. The parameter computations in Saudi Arabia incorporated data from three prominent cities: Jeddah, Al-Medina, Munawara, and Riyadh. Saudi Arabia has received the DQN approach from all countries (Figure 6).

### 4.2 Performance measures for compared models

Considering the context of SA, UK, and TU, the Multi-Head Attention (MHA) method exhibited better performance than the DQN approach, particularly regarding the SMAPE metric. Table 4 summarizes the proposed method's precision on dataset 1, illustrated in Figure 2. The performance of Long Short-Term Memory (LSTM) was comparable to that of MHA. DQN outperformed LSTM in certain countries, while the opposite was the case in others. Neither the MHA nor the CNN models employed the DQN methodology. Table 4 arranges the average SMAPE values from the three countries according to rank. The three deep learning models significantly decreased average SMAPEs compared to the DQN model (0.8).

Regarding SMAPE performance, the MHA and CNN models outperformed the DQN approach. Table 5 presents each model's average percentage of mistakes and identifies the country with the highest ranking. The deep learning models topped the DQN approach model in three countries. Regarding the Mean Absolute Percentage Error (MAPE), the MHA method performed superior to the DQN approach in all three nations. Furthermore, the CNN and LSTM methods surpassed the DQN approach in three out of the four countries. By calculating the mean absolute percentage error (MAPE) scores across all countries, it was determined that deep learning techniques outperformed the DQN methodology.

MHA exhibited the best performance in terms of RMSE among the MAPE indicators. Table 4 shows that both CNN and MHA outperformed the DQN methodology in all three countries studied. The RMSE of the DQN approach exceeded that of the LSTM method.

Nonetheless, the DQN method enabled the acquisition of accurate COVID-19 predictions. A summary of the performance of each suggested approach is provided in Table 5, indicating the reduction of SMAPE, MAPE, and RMSE averages. The contribution of DQN to sequential data assimilation greatly influenced the overall accuracy of these algorithms.

**Figure 7:**
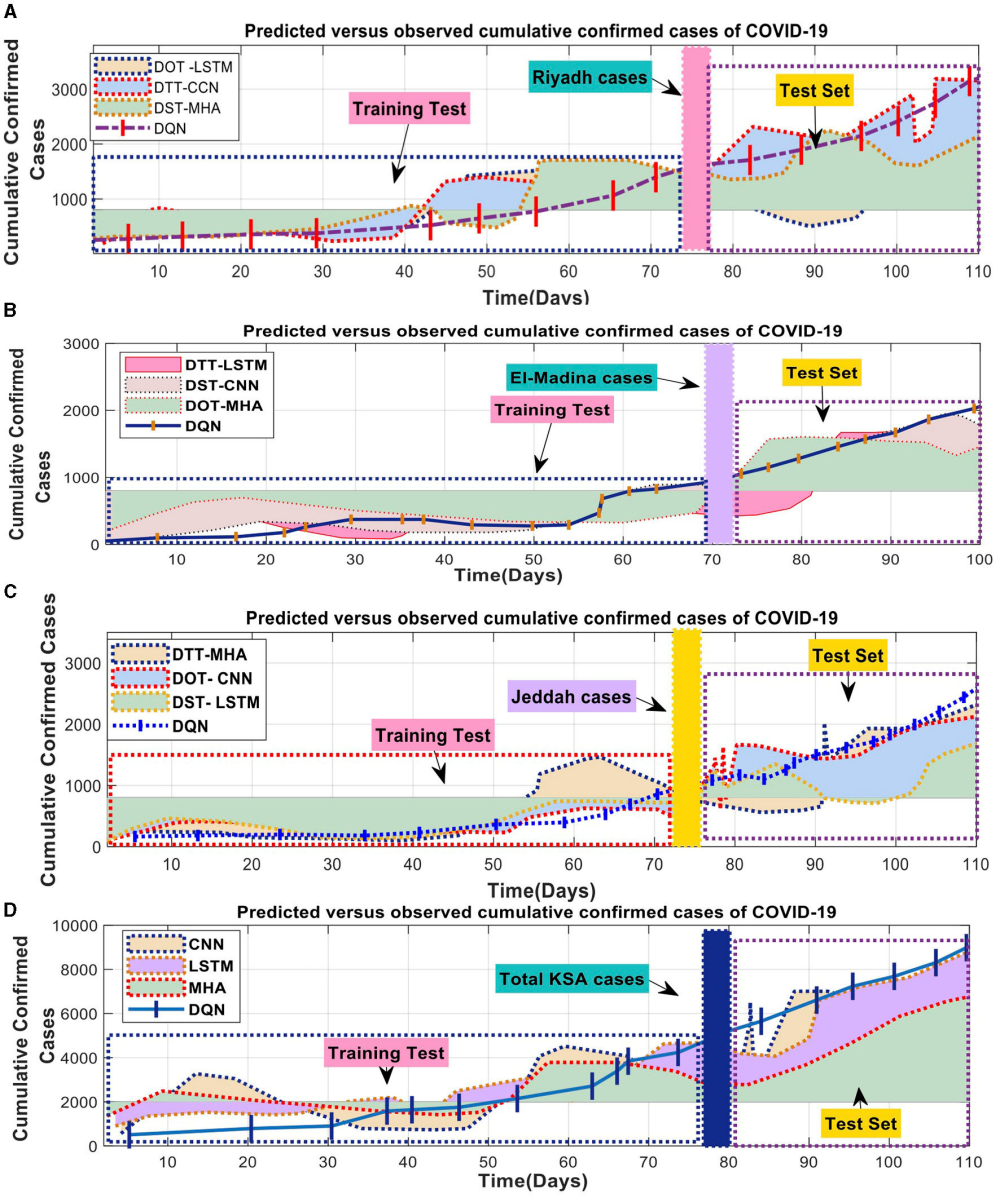
COVID-19 combined the total number of reported cases with the number of expected cases. **(A)** Predicated verse observed cumulative confirmed cases of COVID-19 in Riyadh, **(B)** Predicated verse observed cumulative confirmed cases of COVID-19 in El-Madina, **(C)** Predicated verse observed cumulative confirmed cases of COVID-19 in Jeddah, and **(D)** Predicated verse observed cumulative confirmed cases of COVID-19 in Saudi Arabia.

The best model for predicting COVID-19 cases, spanning several days in three areas of SA, is shown in Figure 7. It shows the Mean Absolute Error (MAE) and Symmetric Mean Absolute Percentage Error (SMAPE). The tested models understood the data patterns and precisely anticipated the observed values. The evaluations confirmed the models' strong execution during the training period. Unfortunately, it was hard for the MHA, LSTM, and CNN models to accurately represent the wide range of initial observations shown in Figures 7A–C. These mathematical models' intricacies in comprehending the underlying patterns were brought to the forefront when interpreting smaller datasets for each state.

**Figure 8:**
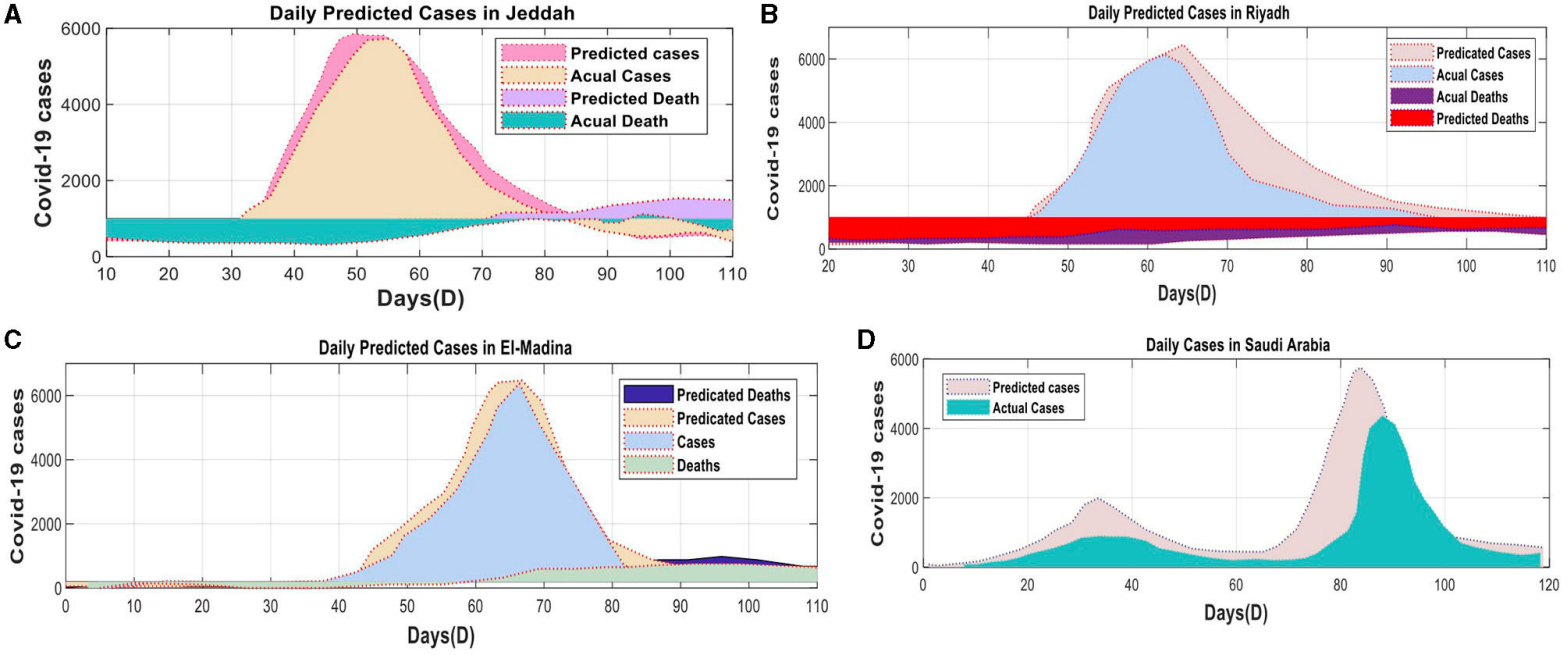
Actual and expected cases and deaths in Saudi Arabia during the preceding 120 days: **(A)** testing cluster—Jeddah, **(B)** testing cluster—Riyadh, **(C)** testing cluster—Madinah, **(D)** testing cluster Saudi Arabia.

In the evaluation, the deep learning model consistently outperforms the DQN strategy concerning the number of deaths and cases. The DQN technique produced consistent linear estimates for death rates and confirmed cases in all counties. In contrast, the superior deep learning models displayed exceptional performance across various domains by accurately capturing linear and nonlinear patterns. Figure 8 exemplifies the deep learning model's ability to identify these patterns precisely, thereby enhancing the overall efficiency of the model. As a result, the proposed model proficiently predicted the COVID-19 time series data.

### 4.3 Performance measures for compared DQN and ARIMA models

**Figure 9:**
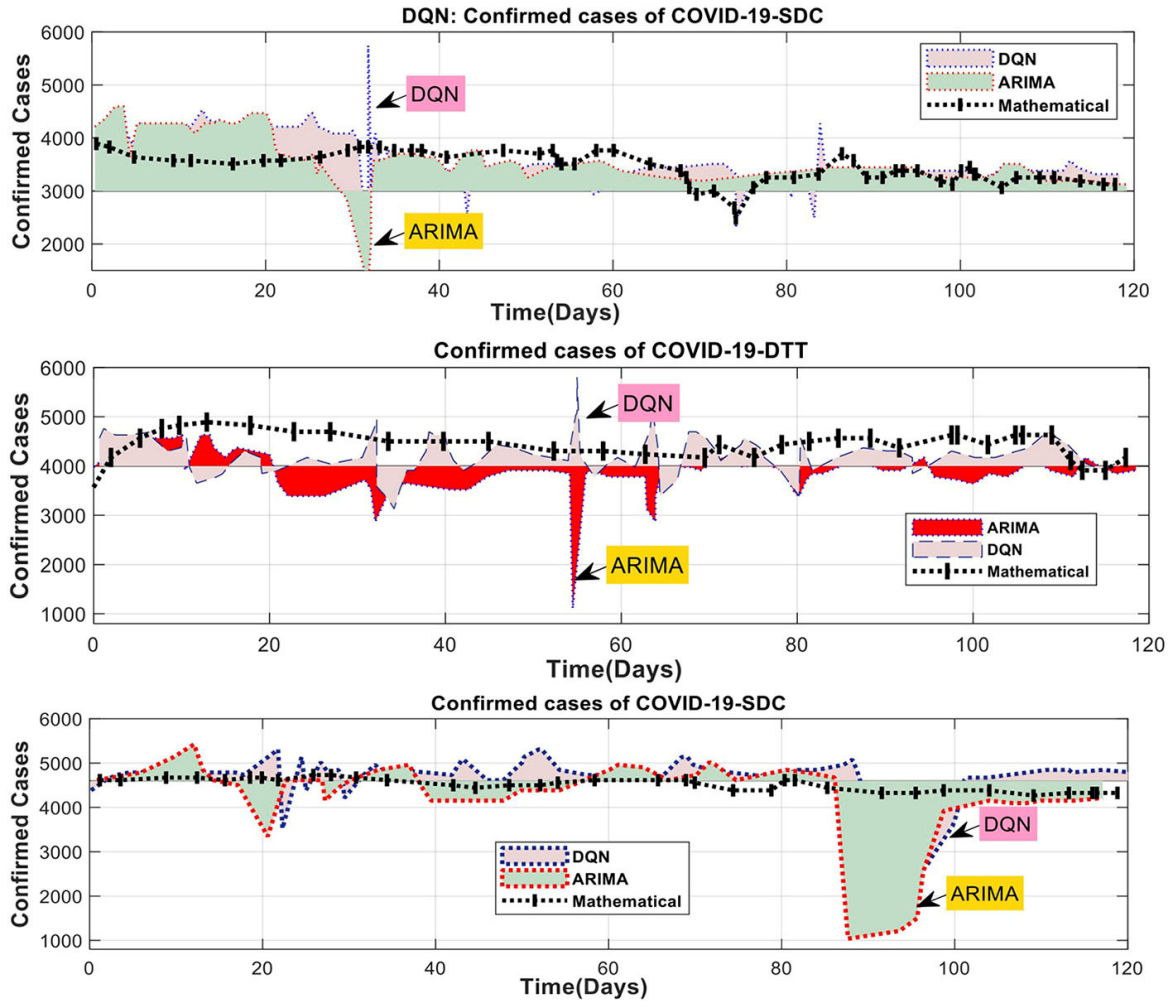
Comparison of DQN modeling results for ARIMA and mathematical residuals.

A machine learning model was used to identify non-linear relationships between the training and test data across three-time intervals. As a result, the residual values were included as the only inputs to the hybrid (ARIMA-mathematical) model. The daily time series of detections was also included as inputs to the DQN model in the ARIMA mathematical model. Figure 9 displays the DQN predictions for the ARIMA fit of the three training tests over three periods. The graph for each period indicates that the ARIMA residuals for the 1-day and three-day tests exhibit frequent fluctuations, while the change for the 6-day test is minimal. The difference in the 6-day test is negligible. The similarity between the projected values and the ARIMA residuals demonstrates the effectiveness of the DQN technique. Predicting the effective reduction of non-linear fluctuations caused by human activities is possible. For example, the data collected over 12 days shows distinct peaks in the DQN model. However, the DQN model must provide more accurate results than the previous ARIMA mathematical model (Figure 9). In contrast, the ARIMA mathematical model systematically reflects the general pattern of the ARIMA residual. The ARIMA mathematical models struggle to accurately capture rapid and substantial changes due to the predominant influence of human actions, as shown in Table 6.

**Table 6:** Errors of three training tests data using ARIMA/mathematical models.

**Models**	**MAPE**	**SMAPE**	**RMSE**	**Days**
DQN	0.1238	0.72425	14.95145	One Day Test
ARINA	0.9876	0.95123	16.75321	Three Days Test
Mathematical	0.2314	0.85213	17.74102	Six Days Test

## 5 Conclusion

This study used Bayesian optimization algorithm (BOA), long short-term memory (LSTM), convolutional neural network (CNN), multi-head attention (MHA), and deep Q-network (DQN) learning methods to guess how many confirmed COVID-19 cases there will be in Saudi Arabia's three most common cities: Jeddah, Riyadh, and Al-Medina Al-Munawara. The performance of the models was assessed utilizing MAPE, MAE, and SMAPE metrics, and stability was evaluated based on out-of-sample error through box plots on the test set. The results suggested that the BOA models precisely predicted COVID-19 case rates in the selected states by understanding the temporal connections within the epidemiological data. The DQN strategy proved especially advantageous for assignments requiring Dynamic Time Warping (DTT) and Dialogue State Tracking (DST). The obtained results proved that the tested models (LSTM, CNN, and MHA) consistently displayed the highest accuracy, with only minor variations in performance across different scenarios. Indeed, the results proved that is essential to exercise caution when interpreting these forecasts, as the data might exhibit atypical variations, and other extraneous factors unrelated to COVID-19 can affect the daily case reports. Combining BOA learning with deep learning algorithms, data enhancement methods on small data sets, and copula functions to calibrate hyperparameters are all possible directions for further research.

The Obtained results proved that the proposed model for the COVID-19 outbreak in SA. The model classifies the population as susceptible, exposed and asymptomatic, infected, and recovered individuals. Firstly, we examine the essential properties of the proposed model, including its positivity and boundedness. Subsequently, we determine the fundamental reproductive number of the model. Our research demonstrates the existence of two distinct equilibrium points in the model: the disease-free equilibrium points and the endemic equilibrium point. The stability of the disease-free equilibrium point can be established when the fundamental reproduction number is less than one, implying the complete eradication of the disease from the population. Further, it becomes evident that the model follows a transcortical bifurcation at the disease-free equilibrium point. This occurs when the bifurcation parameter, representing the disease transmission rate of the infected class, attains a critical value. Our investigation is based on the data collected in Italy between February 15th, 2020, and July 14th, 2020. In the early stages of the outbreak, the virus spread rapidly due to widespread disregard for safety measures among the general population.

Moreover, a significant number of individuals who had contracted the virus arrived in Italy from other countries. Before March 9th, 2020, the disease had already spread extensively throughout the population. The effects of the illness are slowly decreasing due to compliance with measures such as adhering to lockdown, observing home quarantine, regularly washing one's hands, and wearing face masks.

Handling COVID-19 remains challenging, particularly in countries that have previously contained the virus. Our study presents a refined approach to running a non-linear compartmental model that accurately captures the essential dynamic features of COVID-19 while considering limitations. The control design employs a discrete-time iteration of the epidemic model. The system can handle complex situations and logical relationships between model variables and predetermined interventions. A state monitor calculates unobserved variables associated with the current number of patients admitted to the hospital. The work involves numerical simulations of five control scenarios with distinct cost functions and limits. One strategy is to use an output feedback system that includes unclear parameters. The limitations mentioned refer to government measures and tactics to mitigate or exacerbate the issue, depending on the cost function associated with other policy objectives. The findings highlight the need for prompt intervention, careful monitoring of vulnerable groups, and further research to determine strict regulatory measures' exact costs and effects.

## Data availability statement

The original contributions presented in the study are included in the article/Supplementary material, further inquiries can be directed to the corresponding authors.

## Author contributions

AAlh: Conceptualization, Data curation, Writing – original draft. AAlf: Formal analysis, Writing – review & editing. YA: Data curation, Writing – review & editing. HM: Writing – original draft, Writing – review & editing, Data curation. HA: Writing – review & editing, Writing – original draft, Data curation. BS: Formal analysis, Funding acquisition, Writing – original draft, Writing – review & editing.
