# Supplementary material for: Artificial intelligence in healthcare: combining deep learning and Bayesian optimization to forecast COVID-19 confirmed cases
Source: Front Artif Intell. 2024 Jan 11;6:1327355. doi: 10.3389/frai.2023.1327355 (PMC10875994; doi:10.3389/frai.2023.1327355)
Supplement: Supplementary file 1 [file Data_Sheet_1.pdf]

Supplementary Material

Appendix 1: Bayesian Optimization

Bayesian optimization is a widely used method in machine learning to fine-tune hyperparameters. Despite the multitude of technical terms and mathematical formulas associated with it, the fundamental principle is quite simple. The primary aim of this paper is to share knowledge on Bayesian Optimization by providing a concise explanation of basic terminology. I intend to enhance your grasp of Bayesian Optimization in a brief timeframe.

A Primer on Hyperparameter Optimization

This article ensures a comprehensive understanding, starting with a basic summary of the four primary approaches to hyperparameter optimization.

The techniques for exploration encompass manual search, random search, grid search, and Bayesian Optimization.

Bayesian Optimization differs from Random Search and Grid Search by utilizing previous performance to improve search speed, whereas the other two methods remain unaffected by past evaluations. From this perspective, Bayesian Optimization shares similarities with manual search. Consider fine-tuning the hyperparameters of a Random Forest regression model manually. Initially, one would conduct experiments using a defined set of parameters, observe the results, modify a factor, repeat the process, and then compare the outcomes. This iterative approach enables progress to be tracked. Bayesian Optimization works similarly, with the performance of past hyperparameters informing future decision-making. Random Search and Grid Search do not consider the previous version when choosing new hyperparameters for evaluation. Consequently, Bayesian Optimization is a much more efficient technique.

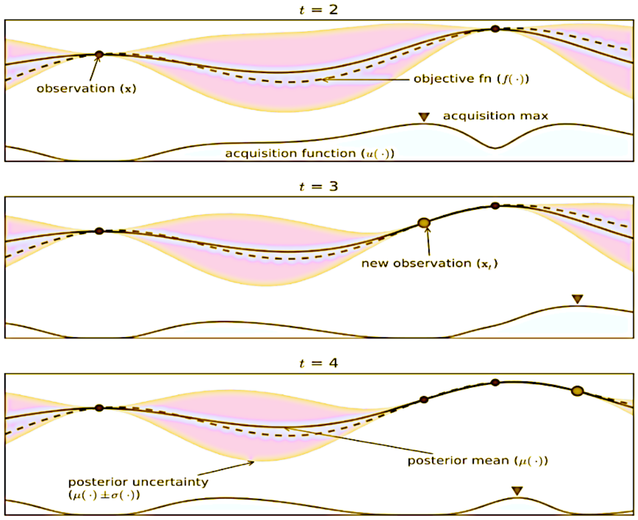

Appendix 3. Bayesian Optimization

Appendix 2: CNN Model

CNN effectively manages machine learning challenges and is employed in this study for predicting time series COVID-19 data. CNNs extract new features from input data through convolution, where a convolution kernel acts as a window to perform operations on the input data and extract new features. These convolutionally derived features are often more discriminative than the original input, enhancing prediction accuracy.

A detailed description of the CNN design methodology is provided in **Appendix 1,** which can be found on this page. This methodology involves three distinct levels: the convolutional level, the pooling level, and the fully connected layer **[49].** Following the necessary pre-processing steps, the convolutional layer extract features from the input time series, the pooling layer further processes the data, and the fully connected layer generates multiple outputs **[32–42].**

4.1.LSTM model

RNNs and other deep learning algorithms excel in analyzing sequences due to their ability to assess temporal behavior. However, they face challenges related to the fade/burst gradient problem, which makes training complex. LSTM, a gated RNN device, effectively addresses this problem, as depicted in **Appendix 1** showcasing the architecture of LSTM blocks **[45-44]**.

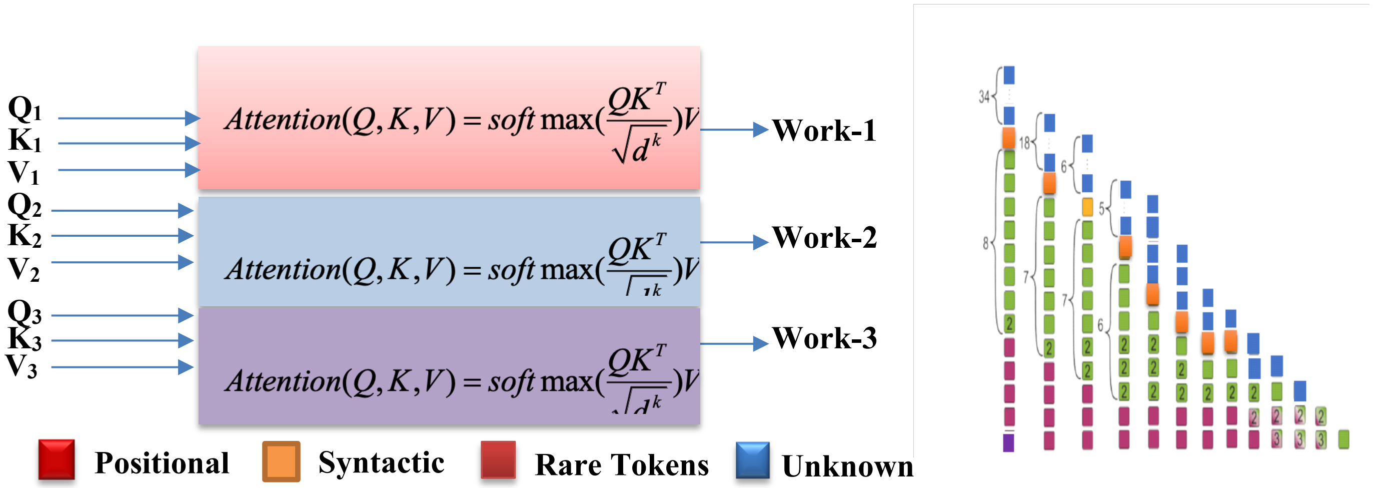

Appendix 2: CNN Model

Appendix 3: LSTM Forecasting model

5. HA model

MHA excels in processing sequences, particularly in the context of natural language processing. The authors have demonstrated in (Aslan et al., 2022) that MHA is well-suited for handling sequential data. In **(Zarzycki and Ławryńczuk, 2022),** the authors presented multi-head models for COVID-19 prediction based on interest. MHA scores enable the learning of diverse interpretations (Fanelli et al., 2022)**.** The MHA consists of three phases: layered attention, streamlined attention, and correlative attention. Once instantiated and configured, the MHA layer determines a precise approach. The flattened layer adjusts the output of MHA, leading to the final result. The fully connected layer generates the outcome. The MHA layer of the model accesses and focuses on the most important data **(**See **Appendix 4)** (Garg et al., 2022)**.** Focus separation is the second stage, involving parallel and independent processing. Equation 5 provides the number of linear observations for the same layer, with each Attention Head (A^H) operating simultaneously.

$$Multihead(Q,K,V) = Concat(head^1, head^2, ..head^n)W^0$$
$$\textbf{Where } A^H : \textbf{Head}^{\textbf{i}} = Attention(QW_i^Q, KW_i^K, VW_i^V) \qquad \textbf{(5)}$$

5.2 Random Forest

Clustering algorithms aim to pair students of similar abilities with those of superior abilities. RF accomplishes this by generating multiple classification trees based on decision trees. Each tree possesses unique characteristics, and these characteristics are used to determine the classification of a new sample. Here is a random selection (Li and Sun, 2022)**:**

a) With **N** sets of training, randomly select **N** super-sampled sample points from the training set.

b) Assuming **M** variables as predictors, at each node of the decision tree, select **m** probability values from all predictor variables, where **m < M**. This selection results in a set of predictor variables. The optimal subgroup division for improved predictions significantly reduces clustering.

c) Repeat steps 1-3 of BOA to create **n** decision trees.

d) All decision trees collectively vote to classify a new sample.

RF avoids mitigates randomness and enhances the decision tree method. Once the quantitative decision trees are established, the data classification result is determined through the majority voting method across all trees, even if it involves a single tree with poor classification. It offers distinct advantages, such as accurate and rapid predictions for large datasets and the ability to handle unbalanced and missing data.

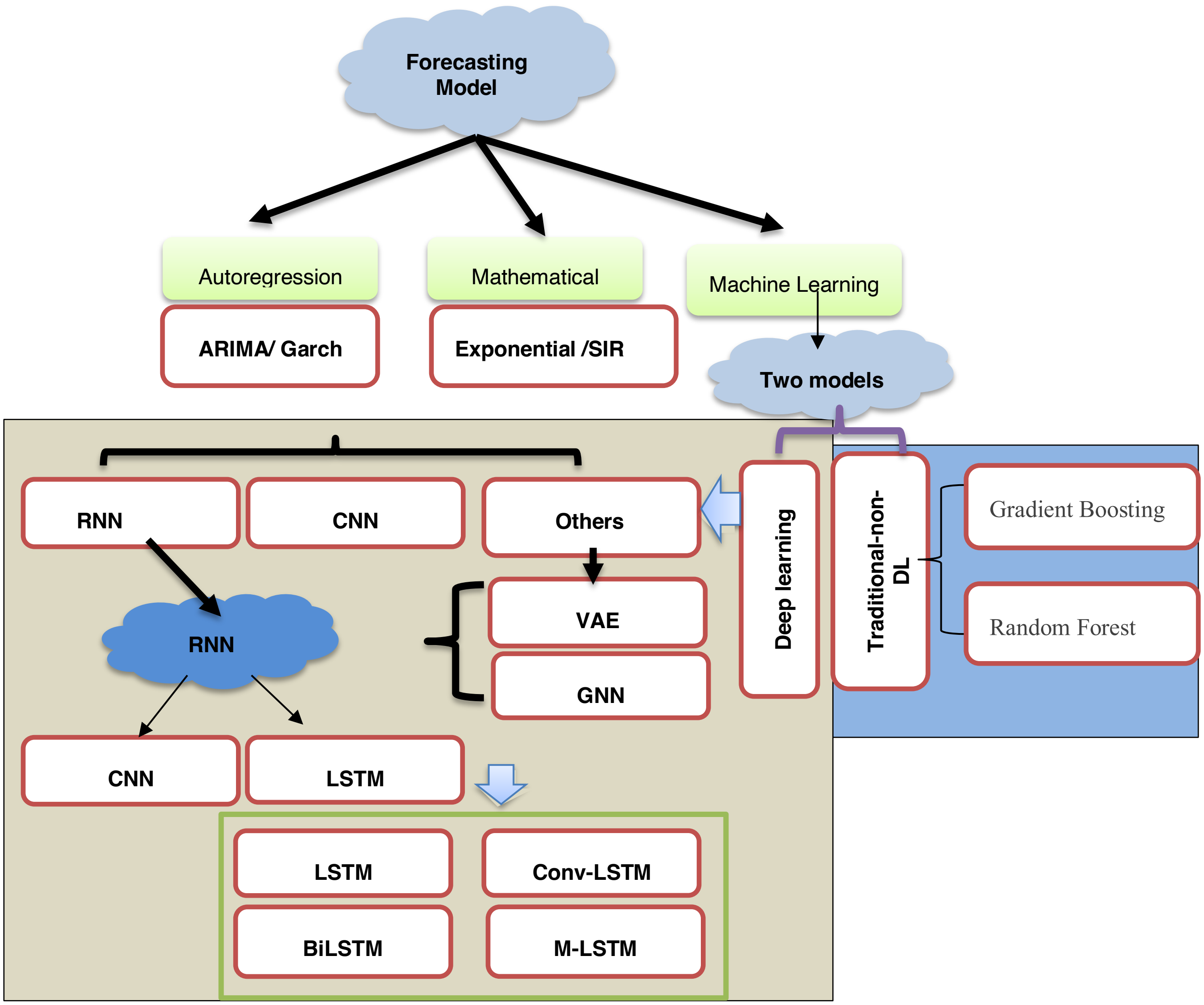

Appendix 3: CNN method: Artificial intelligence system design. The odds of COVID-19 predicted by MSCNN are P **[49].**
